# Outcome impact of individualized fluid management during spine surgery: a before-after prospective comparison study

**DOI:** 10.1186/s12871-020-01092-w

**Published:** 2020-07-22

**Authors:** Lu Che, Xiu H. Zhang, Xu Li, Yue L. Zhang, Li Xu, Yu G. Huang

**Affiliations:** Department of Anesthesiology, Peking Union Medical College Hospital, Beijing, 100730 China

**Keywords:** Orthopaedic surgery, Spine surgery, Individualized fluid management, Stroke volume, Postoperative outcome

## Abstract

**Background:**

Individualized fluid management (IFM) has been shown to be useful to improve the postoperative outcome of patients undergoing major abdominal surgery. A limited number of clinical studies have been done in orthopaedic patients and have yielded conflicting results. We designed the present study to investigate the clinical impact of IFM in patients undergoing major spine surgery.

**Methods:**

This is a before-after study done in 300 patients undergoing posterior spine arthrodesis. Postoperative outcomes were compared between control group implementing standard fluid management (*n* = 150) and IFM group (n = 150) guided by fluid protocol based on continuous stroke volume monitoring and optimization. The primary outcome measure was the proportion of patients who developed one or more complications within 30 days following surgery.

**Results:**

During surgery, patients received on average the same volume of crystalloids (7.4 vs 7.2 ml/kg/h) and colloids (1.6 vs 1.6 ml/kg/h) before and after the implementation of IFM. During 30 days following surgery, the proportion of patients who developed one or more complications was lower in the IFM group (32 vs 48%, *p* < 0.01). This difference was mainly explained by a significant decrease in post-operative nausea and vomiting (from 38 to 19%, *p* < 0.01), urinary tract infections (from 9 to 1%, p < 0.01) and surgical site infections (from 5 to 1%, *p* < 0.05). Median hospital length of stay was not affected by the implementation of IFM.

**Conclusion:**

In patients undergoing major spine surgery, the implementation of IFM was associated with a significant decrease in postoperative morbidity.

**Trial registration:**

ClinicalTrials.gov Identifier: NCT02470221. Prospectively registered on June 12, 2015.

## Background

Intraoperative fluid management is a major determinant of postoperative outcome in various types of surgery [1–3]. Both insufficient and excessive fluid administration are associated with adverse events [4, 5]. In patients undergoing major surgery, individualized fluid management (IFM) has been proposed to tailor fluid administration to individual needs [2, 3]. Multicentre randomized controlled trials (RCTs) and meta-analyses suggest that IFM is beneficial in decreasing postoperative morbidity, shortening hospital length of stay and saving costs [6–11]. Most IFM studies have been conducted in patients undergoing major gastro-intestinal surgery [10–12]. A limited number of studies have been done among patients undergoing orthopaedic surgery and have yielded conflicting results. For instance, in patients undergoing hip fracture surgery, a few studies [13, 14] reported clinical benefits when using IFM, whereas others did not [15, 16]. In addition, it remains unclear whether clinical benefits reported by RCTs are reproducible in real life conditions. The implementation of IFM require education, experience in using hemodynamic monitoring tools, as well as focus and time during the procedure to ensure optimal protocol adherence.

Spine surgery represents a particularly challenging setting for intraoperative fluid management. Prone positioning during the surgery is associated with physiological changes and the surgery itself is associated with significant intraoperative blood loss and postoperative complications [17–19]. Surprisingly, little is known about the impact of IFM in this specific context. Therefore, we designed a before-after comparison study to investigate the impact of IFM implementation on the postoperative outcome of patients undergoing major spine surgery.

## Methods

### Study design and participants

This non-randomized controlled study was approved by the Research Ethics Committee of Peking Union Medical College Hospital and was registered at clinicaltrials.gov (NCT02470221). Written informed consent was obtained from all patients. We studied consecutive adult patients undergoing posterior spine arthrodesis involving more than three vertebral spaces at Peking Union Medical College Hospital. Patients who met any of the following criteria were excluded: emergency surgery, New York Heart Association (NYHA) functional classification class IV or higher, severe aortic regurgitation, inability to cooperate or to sign informed consent.

The study comprised 2 phases. During the first phase (Control group) the use of fluids, vasoactive and inotropic drugs were at the discretion of the anaesthesiologist. During the second phase, hemodynamic management was conducted according to an IFM protocol based on stroke volume monitoring and optimization (IFM group). Before initiating the second phase, members of our clinical staff were trained to become familiar with the hemodynamic monitoring technique and the IFM protocol.

### Anaesthesia and surgical management

General anaesthesia was induced by propofol, fentanyl and rocuronium and maintained with target-controlled infusion of propofol (plasma concentration of 3-5 mg ml/L). After tracheal intubation, patients were ventilated in a volume-controlled mode with a tidal volume of 8 ml/kg. In both groups, a 20 G radial arterial line was inserted for continuous arterial pressure monitoring. The recommendation was to maintain mean arterial pressure ≥ 80% of baseline, with a heart rate ranging between 50 and 100 bpm. Blood transfusion was recommended to maintain haemoglobin > 9 g dl/L.

After anaesthesia induction, all patients were placed in the prone position supported by 4 pads (2 pads under the shoulders and 2 under pelvic sites) to suspend the chest and abdomen from the operation bed. All surgical procedures were performed by the same group of experienced spine surgeons.

### Individualized fluid management

**Fig. 1:**
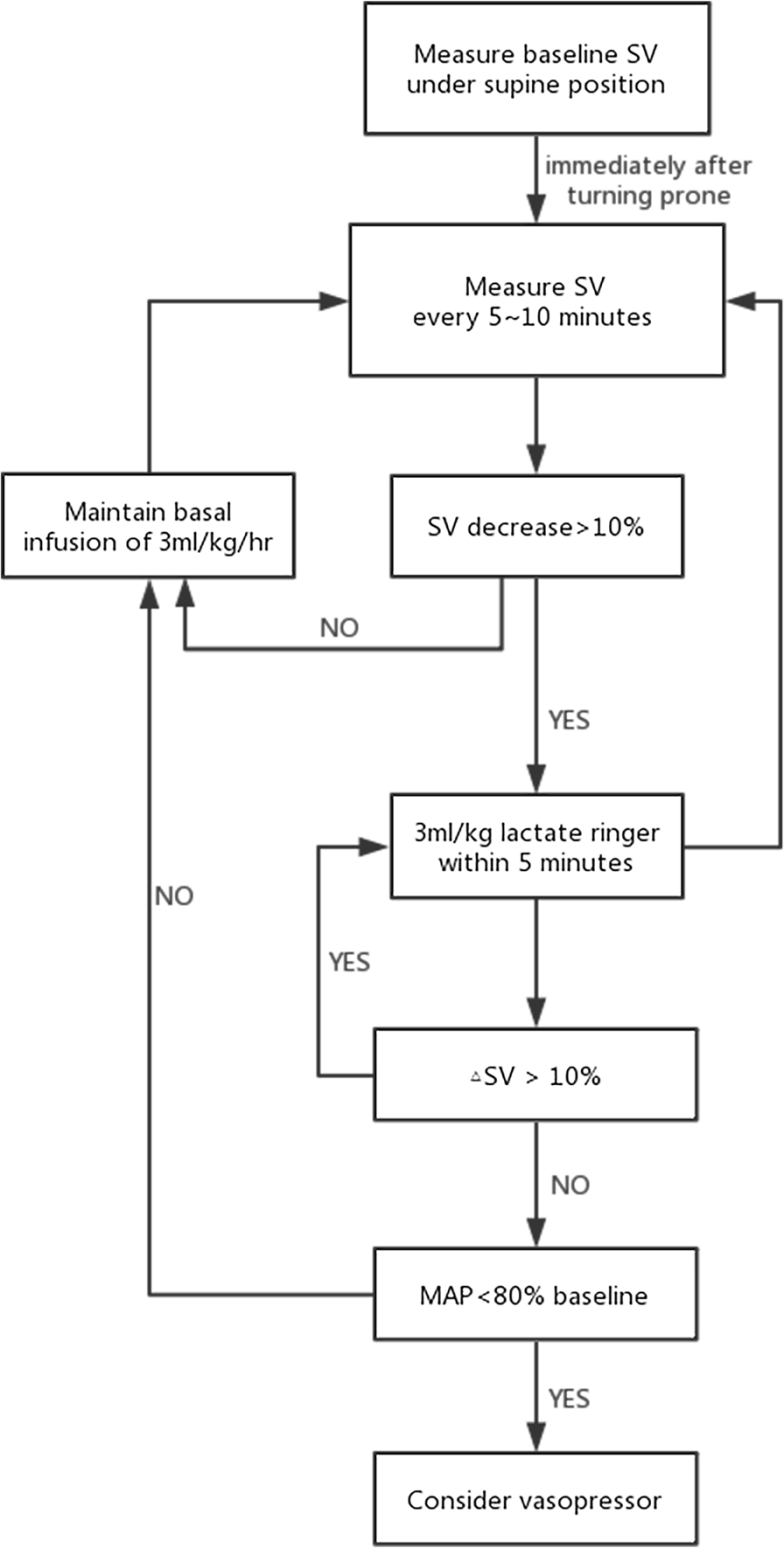
Individualized fluid management (IFM) protocol

In the IFM group (from April 2017) the radial line was connected to the fourth-generation Vigileo/Flotrac system (Edwards Lifesciences, Irvine, CA, USA) enabling continuous monitoring of stroke volume from pulse contour analysis. Fluid maintenance was set at 3 ml/kg/hr. of Ringer’s lactate. Once patients were in the prone position, we started to monitor stroke volume and a bolus of 3 ml/kg of Ringer’s lactate was administered over a 5 min period. The fluid bolus was repeated in responder patients (increase in stroke volume > 10%) until the plateau of the Frank-Starling relationship was reached (increase in stroke volume < 10%). During surgery, additional boluses were given only if stroke volume dropped by > 10% below the plateau value. In case of hypotension (mean arterial pressure < 80% from baseline) in fluid non-responders, vasopressors were recommended. The IFM protocol is summarized in Fig. 1. This fluid management strategy has been used with success in a recent multicentre IFM study [8] and has been recommended in published consensus statements and by national guidelines [20, 21]. Adherence to the IFM protocol was strongly encouraged but not tracked nor quantified.

### Outcome variables

The primary outcome measure was the proportion of patients who developed one or more complications within 30 days following surgery (aka postoperative morbidity). Postoperative complications included gastro-intestinal complications (nausea and vomiting, ileus), infectious complications (urinary tract infection, surgical site infection, pneumonia, bloodstream infection), cardiac complications (cardiac arrest, myocardial infarction, heart failure, arrhythmia requiring pharmacologic treatment, hypotension requiring vasopressor administration, pulmonary embolism, deep venous thrombosis, stroke), and other complications (prolonged mechanical ventilation > 48 h, acute respiratory distress syndrome, acute renal failure according to KDIGO criteria). Diagnosis and management of postoperative complications were undertaken by non-research staff according to our local practice. Postoperative hospital length of stay and mortality were also recorded.

### Sample size calculation and statistical analysis

Based on the 60% postoperative morbidity rate observed in a sample population from our institution, a power analysis indicated that a sample size of around 150 patients in each group was required to show a 25% relative reduction (from 60 to 45%) in postoperative morbidity after IFM implementation, with a power of 0.8 and a type 1 error (α) = 0.05.

Continuous normally distributed variables are expressed as mean ± standard deviation (SD), and non-normally distributed continuous variables are expressed as medians (interquartile ranges). Categorical variables are expressed as numbers and percentages. An independent sample t-test was used to test differences between groups for continuous normally distributed variables, a Chi-square test was used for categorical data to test for differences between groups. When data were not normally distributed, a Mann-Whitney U test was used to analyse differences between groups. The multivariate analysis estimated the association between primary outcomes (composite complication defined as number of patients developing more than one complications) and implementation of IFM controlling for age, sex, American Society of Anesthesiologists (ASA) score, history of hypertension, diabetes mellitus and coronary artery disease using logistic regression modelling. Statistical analysis was performed with SPSS, version 23 (IBM Corp. USA) or STATA, version 14 (Stata Corp. USA). All statistical tests were two-sided, and *p* < 0.05 was considered to indicate statistical significance.

## Results

### Baseline characteristics

Three hundred patients were enrolled between October 2016 and September 2017, 150 patients in the Control group between October 2016 and March 2017, and 150 patients in the IFM group from April to September 2017. Patient characteristics including age, gender, body mass index (BMI), comorbidities and ASA score were not significantly different between the Control and the IFM group (Table 1).

**Table 1:** Baseline patient characteristics

Variables	Control (*n* = 150)	IFM (n = 150)	*p* value
Age (yr)	59.2 (45.4–73.0)	57.9 (40.7–75.1)	0.448
Gender(M/F)	63 (43)	61 (41)	0.725
BMI (kg/m^2^)	25.4 (22.1–28.7)	25.4 (21.5–29.3)	0.971
ASA score(n)			
< =II	144 (91)	141 (94)	
> II	6 (9%)	9 (6%)	1
Comorbidity			
Hypertension	61 (40.7)	66 (44)	0.514
Coronary artery disease	10 (6.7)	10 (6.7)	0.987
Diabetes Mellitus type II	25 (16.8)	21 (14)	0.521
Baseline Hb (g/L)	135 (115–155)	136 (122–150)	0.486
Baseline HR (bpm)	79 (70–88)	77 (68–86)	0.162
Baseline SBP (mmHg)	132 (117–147)	130 (114–146)	0.121
Baseline Creatinine (ug/ml)	67 (54–80)	68 (49–87)	0.657

Data are presented as mean ± SD, or absolute numbers (percentage). IFM: Individualized fluid management; BMI: Body mass index; ASA: American Society of Anesthesiologists; Hb: Haemoglobin; HR: Heart rate; SBP: Systolic blood pressure

### Intraoperative fluid management

During surgery, both groups (Control and IFM) received the same average amount of crystalloid and colloids (Table 2). Estimated blood loss and urine output were comparable as well (Table 2). As a result, the intraoperative fluid balance was not different between Control and IFM patients (Table 2).

**Table 2:** Intraoperative data

Variable	Control (*n* = 150)	IFM (*n* = 150)	*P* value
Operation time (min)	193 (156,225)	184 (153,220)	0.404
Infused crystalloids (ml/kg/h)	7.4 (5.1–9.7)	7.2 (4.6–9.8)	0.398
Infused colloids (ml/kg/h)	1.6 (0.3–2.9)	1.6 (0.3–2.9)	0.893
Cell saver use, n(%)	80 (53.3)	94 (62)	0.129
RBC transfusion, n(%)	20 (13.3)	29 (19.3)	0.159
Urine output (ml/kg/h)	2.8 (1.6–4.4)	3.2 (1.1–5.3)	0.121
Estimated blood loss (ml)	470 (53–1357)	529 (140–918)	0.062
Intraoperative fluid balance (ml/kg/h)	5.1 (2.2–8.0)	5.1 (2.2–8.0)	0.904
Vasopressor, n(%)	20 (13.33)	7 (4.67)	0.009
Phenylephrine, n(%)	2 (1.33)	1 (0.67)	0.391
Ephedrine, n(%)	19 (12.67)	6 (4.00)	0.07
Phenylephrine continuous infusion, n(%)	0 (0)	1 (0.67)	0.500

Data are presented as mean ± SD, median (interquartile range), or absolute numbers (percentage). IFM: Individualized fluid management; RBC: Red blood cells

### Postoperative outcomes

Overall, less patients developed one or more complications (32 vs 48%) in the IFM group (Table 3). The proportion of patients who developed postoperative nausea and vomiting (PONV), urinary tract and surgical site infections was significantly lower in the IFM group than in the control group (Table 3). Hospital length of stay was comparable in both groups (Table 3). None of the 300 patients died within the 30 days following surgery. Upon multivariate analysis (Table 4) implementation of IFM demonstrated statistically significant associations with postoperative composite complications after controlling for age, sex, ASA score, BMI and comorbidities (OR = 0.481, 95% CI 0.295 to 0.786, *P* = 0.003).

**Table 3:** Postoperative outcome data within 30 days

	Control (*n* = 150)	IFM (*n* = 150)	*P* value
**PRIMARY OUTCOME**			
Patients with one or more complications, n (%)	72 (48)	48 (32)	0.005
**COMPONENT OF COMPLICATIONS**			
**GASTRO-INTESTINAL COMPLICATIONS**			
PONV, n (%)	55 (38)	29 (19)	0.001
Ileus, n (%)	1 (1)	0 (0)	1
**INFECTIOUS COMPLICATIONS**			
Urinary tract infection, n (%)	14 (9)	1 (1)	0.001
Surgical site infection, n (%)	8 (5)	1 (1)	0.017
Pneumonia, n (%)	3 (3)	3 (2)	1
Blood stream infection, n (%)	1 (1)	0 (0)	1
**CARDIAC COMPLICATIONS**			
Cardiac arrest, n (%)	0 (0)	0 (0)	1
Myocardial infarction, n (%)	0 (0)	1 (1)	1
Heart failure, n (%)	0 (0)	0 (0)	1
Arrhythmia, n (%)	0 (0)	1 (1)	1
Hypotension, n (%)	2 (1)	0 (0)	0.498
Pulmonary embolism, n (%)	0 (0)	1 (0.7)	1
Deep venous thrombosis, n (%)	2 (1.3)	0 (0)	0.498
Stroke, n (%)	1 (0.7)	0 (0)	1
**OTHER COMPLICATIONS**			
Prolonged mechanical ventilation, n (%)	1 (1)	0 (0)	1
Acute kidney injury, n (%)	11 (7)	20 (13)	0.1
ARDS, n (%)	0 (0)	0 (0)	1
**SECONDARY OUTCOME**			
ICU admission, n (%)	12 (8)	13 (9)	0.834
Postoperative hospital length of stay (days)	14 (12–18)	14 (10–18)	0.576
Mortality, n (%)	0 (0)	0 (0)	1

Data are presented as mean ± SD, median (interquartile range), or absolute numbers (percentage). IFM: Individualized fluid management, ICU: Intensive care unit, ARDS: Acute respiratory distress syndrome, PONV: postoperative nausea and vomiting

**Table 4:** Multivariate analyses of association of IFM and primary outcome

Model	Variable	Odds ratio	95% CI	*P* value
Crude	IFM	0.510	0.319–0.815	0.005
Adjusted	IFM	0.481	0.295–0.786	0.003
	Age	0.999	0.982–1.017	0.626
	Sex	2.850	1.712–4.743	< 0.001
	BMI	1.044	0.970–1.125	0.252
	ASA score	0.584	0.179–2.008	0.962
	Hypertension	0.587	0.357–0.966	0.360
	Diabetes mellitus	1.485	0.731–3.020	0.269
	Coronary artery disease	3.003	0.957–9.416	0.089

*IFM* Individualized fluid management, *BMI* body mass index, *ASA* American society of anaesthesiologists

## Discussion

Our study demonstrated that the implementation of IFM for patients undergoing major spine surgery was possible and effective in our institution. Indeed, it was associated with a significant reduction in postoperative morbidity.

Our findings are consistent with the results of several RCTs and meta-analyses which have demonstrated that IFM is susceptible to improve the postoperative outcome of patients undergoing major surgery, and in particular to decrease PONV [22, 23] urinary tract and surgical site infections [6, 24, 25]. However, the beneficial effects of IFM have been questioned in patients undergoing orthopaedic surgery. In patients undergoing hip fracture surgery, two small studies (< 100 patients) have reported shorter times to being declared medically fit for discharge when using IFM [13, 14]. But more recent and larger studies did not confirm the clinical benefits of IFM in this surgical patient population [15, 16]. Significant reductions in postoperative complications with IFM have also been reported in a small RCT done in 40 patients undergoing primary hip surgery [26] and in a larger study of patients undergoing hip revision [27]. Peng et al. [28] observed a significant improvement in gastro-intestinal function with IFM in a RCT of 80 orthopaedic patients, where 34 of them underwent spine surgery. Therefore, our study is the largest evaluation of IFM in orthopaedic patients and the first one with a focus on spine surgery.

Interestingly, total intraoperative fluid volumes were not significantly different between the Control and the IFM group. At first sight, it may appear somewhat surprising to observe differences in postoperative outcome without observing differences in the volume of fluid administered during surgery. Actually, this finding is consistent with the results of recent multicenter studies [8] and meta-analyses [12]. Indeed, it has been hypothesized that the individualization of fluid therapy is effective through timely replenishment of fluid for patients who are fluid responders and avoidance of fluid overload for those who are not [10]. With the guidance of IFM protocol, fluid responders are more likely to receive more fluid and non-responders more likely to receive less. This may explain why the average volume of fluid was comparable between groups.

The decrease in postoperative complications was not associated with a significant decrease in hospital length of stay. Several reasons could explain this finding. First, the implementation of IFM was associated with a significant decrease in minor complications, which are less likely to impact length of stay than major complications. Second, hospital discharge depends not only on postoperative complications but also on cultural and logistic factors such as the agreement from the patient or their family, as well as the availability of a structure for re-education. In this respect, several IFM studies have reported a significant decrease in postoperative complications that was not associated with a significant reduction in hospital length of stay [7, 29].

Our study has limitations. Because it was not an RCT, we cannot claim causality between IFM implementation and the observed decrease in postoperative morbidity [30]. Another potential disadvantage of this study design is the risk of imbalance between groups. Luckily, given the size of our study (300 patients), there was no visible difference at baseline between the Control and the IFM groups. Randomized controlled trials are essential research tools with strong internal validity but low generalizability to real life conditions [30–32]. In contrast, before after comparison studies provide valuable data regarding the effect of an intervention in real-life conditions, rather than under the stringent conditions of a RCT [30, 31]. Similar study design has been used in several landmark studies which had a significant impact on quality of surgical and critical care [33, 34]. In addition, several quality improvement programs have confirmed the clinical value of IFM in patients undergoing major abdominal surgery [35–38]. However, to the best of our knowledge, our study is the first real life evaluation of IFM in patients undergoing spine surgery. We did not use tracking tools or target screens to quantify and optimize compliance to our IFM protocol. We are well aware that such tools are now available on modern hemodynamic monitoring systems [39] but they were not on our Vigileo monitor. In addition, diagnosis of postoperative complications was carried out by non-research staff according to our institutional practice, so that there was no official definition for each complication during the study period. Finally, monitoring equipment would increase costs which may be a barrier to hospital adoption [40–42]. Cost-effectiveness is an important consideration [42, 43]. Unfortunately, in this study we were unable to assess the impact of IFM implementation on health care costs.

## Conclusions

In patients undergoing major spine surgery, the implementation of IFM was associated with a significant decrease in postoperative complications that, however, did not impact hospital length of stay. Further studies are required to assess the economic impact.

## Data Availability

The datasets used and/or analysed during the current study are available from the corresponding author on reasonable request.

## Abbreviations

IFM: Individualized fluid management
RCTs: randomized controlled trials
ASA: American Society of Anesthesiologists
NYHA: New York Heart Association
PONV: postoperative nausea and vomiting
Hb: Haemoglobin
HR: Heart rate
SBP: Systolic blood pressure
ICU: Intensive care unit
ARDS: Acute respiratory distress syndrome
